# Quality of emergency medical care in Gondar University Referral Hospital, Northwest Ethiopia: a survey of patients’ perspectives

**DOI:** 10.1186/1471-227X-14-2

**Published:** 2014-01-23

**Authors:** Belaynew Wasie Taye, Mensur Ousman Yassin, Zemene Tigabu Kebede

**Affiliations:** 1 Department of Epidemiology and Biostatistics, Bahir Dar University, Bahir Dar, Ethiopia; 2 Department of Surgery, University of Gondar, Gondar, Ethiopia; 3 Department of Paediatrics, University of Gondar, Gondar, Ethiopia; 4 Operational Research Advisor, Amhara Regional Health Bureau, Bahir Dar, Ethiopia

**Keywords:** Emergency care, Quality, Patient satisfaction, Gondar, Northwest Ethiopia

## Abstract

**Background:**

Ethiopia has fairly good coverage but very low utilization of health care services. Emergency medical care services require fast, correct and curious services to clients as they present with acute problems. In Ethiopia and Gondar in particular, the quality of emergency medical care has not been studied. The main aim of this study was to assess the disease profile and patients’ satisfaction in Gondar University Referral Hospital (GURH).

**Methods:**

A facility based cross-sectional study was conducted among patients visiting GURH for emergency care. Ethical clearance was obtained from the Institutional Review Board of University of Gondar. Patients were selected by systematic random sampling, using patient flow list in the day and night emergency services. Data were collected using a standard Press Ganey questionnaire by BSc health science graduates. Data were entered in to Epi Info 3.5.3 software and exported to SPSS version 20.0 for windows for analysis.

**Results:**

A total of 963 patients (response rate = 96.8%) were studied. The mean (+ s.d.) age of patients was 28.4 (+17.9) years. The overall satisfaction using the mean score indicates that 498 (51.7%) 95%CI: (48.4% - 54.9%) were satisfied with the service, the providers and the facility suitability whereas 465(48.3%) 95%CI: (45.1%- 51.6%) were not satisfied. Seven hundred and six (73.3%) 95%CI: 70.4%-76.1%, patients reported that they have been discriminated or treated badly during the service provision in the hospital. OPD site visited (p < 0.0001), visiting days of the week (P < 0.049), medical condition on arrival (P < 0.0001), degree of confidence in the hospital (AOR = 1.9, 95%CI: 1.1, 3.1), reported discrimination/bad treatment of patients with service (AOR = 0.4, 95%CI: 0.2, 0.7), were significantly associated determinants of patient satisfaction.

**Conclusions:**

Non-communicable disease emergencies like injuries and cardiovascular diseases are common. There is a low level of patient satisfaction related to lack of confidence in the hospital for treatment, discrimination towards patient care, and under and delayed treatment of patients who were not in serious medical conditions. Hospitals shall prepare themselves to address the increasing challenge of non-communicable disease emergencies. It is important to revise the service delivery in the emergency department to improve staff courtesy and politeness, commitment, reduce discrimination and bad treatment and proper triage of emergencies at all points of care to increase patient satisfaction giving emphasis to earlier working days.

## Background

Ethiopia has good health coverage with about 89% physical access or reachability, but unacceptably low utilization at 29%. Cognizant of this fact, the Ethiopian Federal Ministry of Health has stated that universal access to emergency service will be provided to all citizens [1,2]. The low rates of utilization of care entail the absence of basic service capacity standards, affordability, weak referral systems and quality of services [1].

With a growing focus on disease control with emergency care and non-communicable diseases, [3,4] medical emergency care is becoming a medical specialty in many developed countries while managed sporadically in the developing countries [5]. The major reasons for emergency OPD visits are gastroenteritis/diarrhea, lower respiratory infections, malaria, ischemic heart disease, septicemia, and injuries [6,7]. Public violence among men and domestic violence to women that are commonly seen in young people are also important causes of emergency department visits [8].

Currently in Ethiopia, cardiovascular admissions- notably due to ischemic heart disease- have risen in the last two decades and it is reported that there are no referral facilities within 100 km [9]. The median waiting time for patients is about 8 min (from 0 to 3 h 59 min), and the median therapeutic time was 56 min (from 5 min to 16 h 19 min) [7].

Persons who experience pain and other symptoms as life threatening [10], men and older patients, persons who are triaged for the more advanced illnesses, those nearer to the hospital, patients with psychiatric disorders, and asthmatics visit emergency clinics more frequently than the normal population [11-14]. In some cases, patients report more often at the beginning of the week than on the weekends [7]. Perceived urgency of disease, a younger population, females, non-attended patients during the day time, a longer duration of the illness, and non-traumatic injuries are the group of patients who visit the emergency OPD for a non-urgent care [15].

Patient satisfaction in emergency care is a challenging experience.

The patients seek high quality care but there is an absence of well-organized facilities and experienced, dedicated staff and this leads to patient dissatisfaction. This dissatisfaction is a major problem in emergency medical care [16]. The level of satisfaction in emergency care ranges from as low as 2% in Pakistan [17] and 63% in Iran [18] to as high as 99.5% in United Sates [16].

The major reasons for the dissatisfaction are interpersonal communications [16], system problems including inadequately equipped facilities, no budget allocates for emergency departments and a lack of critical supplies which are needed in emergency situations [17]. Other determinants of satisfaction are physicians’ and nurses’ communication with patients, security guards’ courtesy and communication, the mean waiting time, and the occurrence of unscheduled events which delayed care [18,19].

Classification of patients’ degree of problems for priority care, and training of staff on emergency care improved patient care and ensured better patient satisfaction [20,21].

Quality of care is a corner stone in an organizations’ goal. Currently, the Ministry of Health is expanding emergency care to the needy populations. The Gondar University Referral Hospital has a goal of improving satisfaction of patients [22]. There is lack of evidence that assesses the quality of care in emergency units among hospitals in the Amhara Regional State. This study assessed the disease profile, level of patient satisfaction and determinants of quality emergency care in a tertiary hospital in Northwest Ethiopia.

## Methods

### Study design

This was a hospital based cross-sectional study that assessed disease profile and quality of service among patients presenting to emergency department of GURH.

### Setting

The study was conducted in GURH. This is a tertiary teaching hospital serving about 5 million people. The hospital has 518 beds and sees between 350 to 400 patients each day and between 100-120 emergency patients. The hospital has four emergency suites with a triage unit for distribution. It is staffed by about 270 nurses and 150 physicians. All adult cases pass through the triage unit of the hospital before seeing doctors except for children who go directly to the pediatric clinic.

### Study population

The study population was all cases reporting to the emergency department with any emergency problem and presenting to all the OPDs. Patients presenting to the follow up clinic or for regular services and women presenting to the hospital for normal delivery services were excluded.

### Sample size and sampling techniques

The sample size was determined using single population proportion formula: $n=\frac{{Z^{2}}_{\alpha /2}\times p\left(1-p\right)}{W^{2}}$

The assumptions were, P = 63% (the proportion of good quality of emergency medical care measure by patient satisfaction from Iranian study [18]; Z = 1.96, (the value of standard normal distribution at 95% confidence level, w = 3%, (marginal error) giving a sample size of 995 patients.

The study participants were selected by a systematic random sampling technique where the first case was identified among the 1-4 lists of patients presenting first at the start date of data collection using a lottery method. Thereafter, every 4^th^ subject at each section of the hospital’s emergency departments was interviewed. Patients coming in both during the day and night hours were included. In the case where a patient was in distress and could not be interviewed, the care takers of the patients were consulted. The severity of patients was determined subjectively by clinicians.

### Study variables

The outcome variable was quality of emergency care measured in terms of patient satisfaction. The explanatory variables included socio-demographic characteristics, the OPD sites, the day of the week, medical condition, perception about the hospital care, history of admission, time of arrival, the patient’s perception of service, and courtesy of hospital staff.

### Definitions

*Medical emergency* was defined as a condition wherein patients presented with acute illness /accident within 48 hrs and chronic patients with acute exacerbations within 48 hrs, unstable patients- such as patients with grossly abnormal vital signs or unconsciousness, and metabolic disturbances. *Quality of emergency care* was perceived satisfaction of care by emergency patients. Patient satisfaction was defined as the feelings of pleasure or disappointment as a result of a rendered service with a comparison of the performance of the institution’s care against the expectations of the patient [23]. *Patient satisfaction* was measured by a Likert scale of 20 questions and was graded as very dissatisfied, dissatisfied, fair/indifferent, satisfied and very satisfied. Those scoring the mean or below were considered as dissatisfied while a score above the mean was labeled as satisfied.

### Data collection instrument and procedures

Data were collected by a standard modified 20 items Press Ganey questionnaire developed in English, translated to Amharic and back translated to English by different person to check for consistency. A pre-test was conducted on 20 patients in the Gondar Polyclinic before the main and the instrument was amended accordingly.

An exit interview was conducted after patients were examined and treated. To avoid social desirability bias, data collection took place in a private area. If a patient was unconscious or in distress, care takers gave consent and were interviewed.

Data collectors were graduate nurses, health officers and environmental health technicians who were not working in the emergency department. Training was provided on the data collection techniques and utilization of the study tool for one day. There was daily supervision of data collection by the investigators. The completed questionnaires were checked for completeness and accuracy every day. Confidentiality of information was assured through use of the anonymous questionnaire. A code was used to identify the patient to avoid repeat interviews.

### Statistical analysis

Data were entered in to the Epi Info Version 3.5.3 statistical package by data entry clerks, then cleaned and exported to SPSS version 20.0 for windows for analysis by investigators. Descriptive statistics were calculated to present socio-demographic characteristics, disease profiles, and levels of satisfaction. Bivariate and multiple logistic regression analyses were used to determine the association of different factors with satisfaction. *P-values* less than 0.05 or 95% CI not including the null value were considered as statistically significant.

### Ethical issues

Ethical clearance was obtained from Institutional Review Board of the University of Gondar. A letter of permission was obtained from the chief executive officer of the hospital. Data collection resumed after informed consent was obtained from each patient or guardian if aged less than 18 years. For patients who were unable to give consent due to age or serious illness, caretakers and guardians gave the consent and were interviewed. To ensure confidentiality, an anonymous questionnaire was used and the interviews were conducted in a private area. All patients, care takers or guardians had the right to withdraw at any point during data collection without any consequences to the quality of service.

## Results

### Socio-demographic characteristics of patients

A total of 963 patients were included in the study with a response rate of 96.8%. The other 3.2% participants were either non-response or excluded due to incomplete. The mean age of patients was 28.4 (+17.9) years. Children under the age of 15 years accounted for 20.6 percent of the participants with 9.2% under the age of five years. Elderly patients accounted for 4.6%. There were comparable numbers of males (48.5%) 95%CI: 45.5%-51.7%, and females (51.5%) 95%CI: 48.3%-54.5%. Nearly two-thirds (60.7%) 95%CI: 57.7%-63.9%, of the patients were from rural areas and 271 (28.1%) were housewives by occupation followed by students comprising 21.3% of all patients. Most of the patients (81.4%), 95%CI: 80.1%-86.4%, arrived during the morning (AM) hours (Table 1).

**Table 1:** Socio-demographic characteristics of patients at the emergency departments in Gondar University Referral Hospital, Northwest Ethiopia, May 2012

**Characteristic**	**Number of patients**	**Percent**
**Age of patient**		
<5	89	9.2
5-14	110	11.4
15-24	249	25.9
25-34	211	21.9
35-44	108	11.2
45-54	102	10.6
55-64	50	5.2
65+	44	4.6
**Sex of patient**		
Male	467	48.5
Female	496	51.5
**Residence of patient**		
Rural	585	60.7
Urban	378	39.3
**Occupation of patient (n = 837)**		
Merchant	34	3.5
Farmer	182	18.9
Student	205	21.3
Housewife	271	28.1
Government employee	58	6.0
Daily laborer	36	3.7
Other	51	5.3
**Days of visit**		
Monday	115	11.9
Tuesday	195	20.2
Wednesday	190	19.7
Thursday	158	16.4
Friday	113	11.7
Saturday	96	10.0
Sunday	96	10.0
**OPD site visited**		
Medicine	416	43.2
Obstetrics/gynecology	125	13.0
Pediatrics	166	17.2
Surgery	232	24.1
Oral health, psychiatry, eye	24	2.5
**Time of arrival at OPD/emergency unit**		
Morning	784	81.4
Afternoon/evening	179	18.6

### Profile of diseases and general medical condition of patients

**Figure 1:**
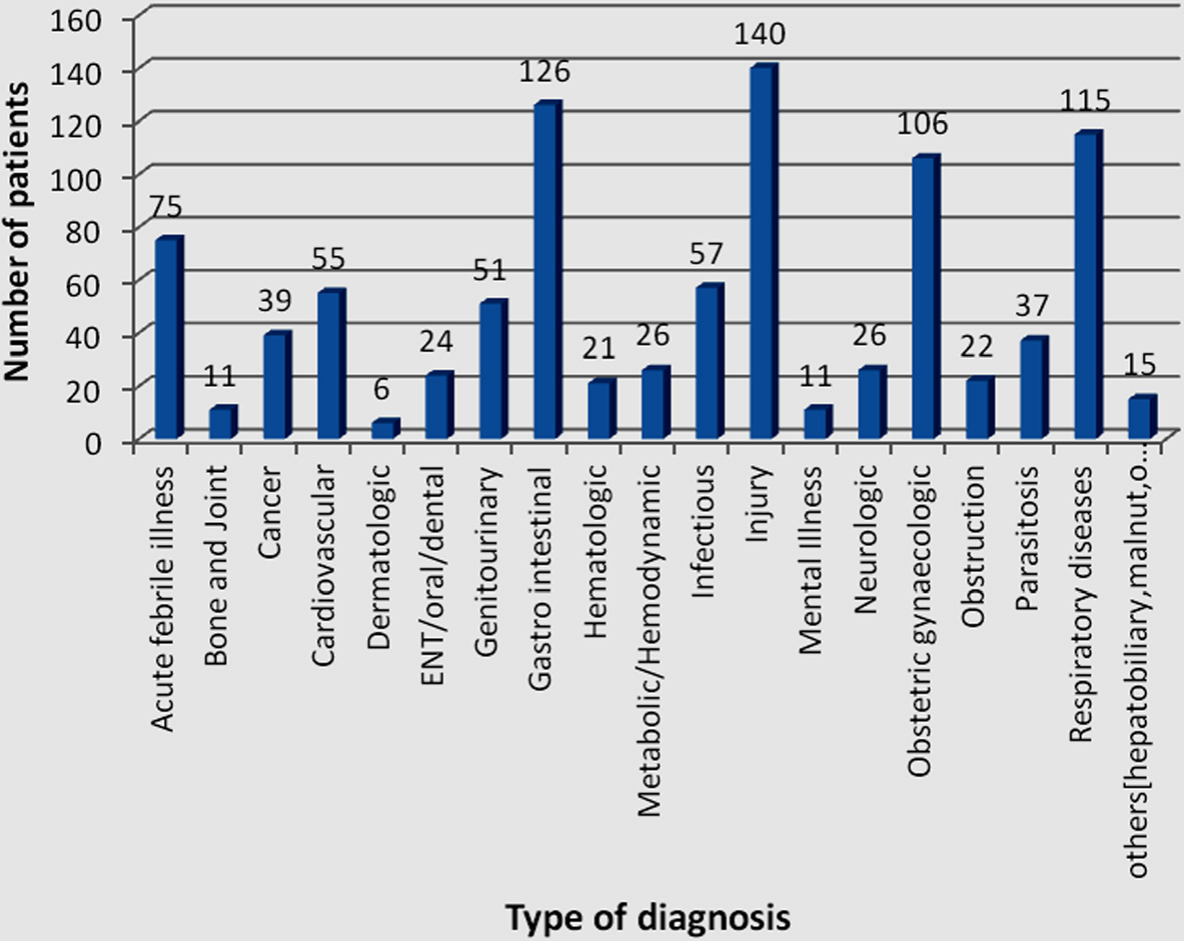
Disease profile of patients at emergency departments in Gondar University Referral Hospital, May 2012.

The most common diagnosis in the emergency OPD was injury seen in 140 (14.5%), 95%CI: 12.4%-16.8%, patients. Gastrointestinal disorders took the next greater share with 126 (13.1%), 95%CI: 10.9%-15.5%, patients followed by respiratory diseases 115 (11.9%), 95%CI: 9.4%-14.6%, and obstetric/gynecologic emergencies (11.0%), 95%CI: 8.9%-13%. Cardiovascular problems were also significant and were observed in 55 (5.7%) of the patients. Cancers of any form were also observed in 39 (4%) of the patients (Figure 1).

At the time of arrival at the emergency OPD, 422 (43.8%), 95%CI: 40.6%-46.8%, patients were very sick or in critical condition while a similar proportion, 416 (43.2%), 95%CI: 40.1%-46.2%, were moderately sick. A total of 125 (13.0%), 95%CI: 10.7%-15.2%, of the patients were in good condition. Five hundred eight (52.8%) patients were managed in the emergency unit while the rest were either admitted, 452 (46.9%) to the respective wards or referred, 3(0.3%) to another facility. The patient’s stays in the emergency department ranged from 1-2 hours (29.3%), 95%CI: 25.2%-33.7%, to as long as 24 hours or more (17.5%) 95% CI: 13.8%- 21.1%. The mean duration of the stay in the emergency department was 16.9 hours (Table 2).

**Table 2:** Medical conditions of patients at emergency departments in Gondar University Referral Hospital, Northwest Ethiopia, May 2012

**Medical characteristic**	**Number of patients**	**Percent**
**Presence of any past illness**		
Yes	580	60.2
No	383	39.8
**General condition of patient on arrival**		
Good condition	125	13.0
Somewhat sick	416	43.2
Very sick	422	43.8
**Subsequent management decision**		
Managed in emergency room	508	52.8
Admitted	452	46.9
Referred	3	0.3
**Emergency visit before**		
Yes	538	55.9
No	424	44.0
I don’t know	1	0.1
**Duration of stay in emergency department (n = 508)**		
1-2 hours	149	29.3
3-6 hours	124	24.4
7-12 hours	61	12.0
13-21 hours	85	16.7
>/=24 hours	89	17.5
**History of admission to hospital**		
Yes	104	10.8
No	857	89.0
I don’t know	2	0.2
**Previous chronic illness**		
No	672	69.8
Yes	291	30.2
High blood pressure	42	4.4
Cardiac problem	116	12.0
Cancer	10	1.0
Diabetes	25	2.6
Anxiety	70	7.3
Obesity	3	0.3
Asthma	25	2.6

### Health service related characteristics of patients

One hundred sixty-nine (17.6%), 95%CI: 15.4%-20.0%, patients cancelled their emergency room visit while there was a perceived reason. The main reasons for cancelling their visit were a lack of money, 92 (54.4%), and in 67 (39.7%) of them because of a mix of reasons including not trusting accessibility of service, family problems and a preference for traditional medicine.

Regarding confidence in getting good service from the hospital, 582(60.4%) were very confident, 268 (27.8%) confident, 40 (4.2%) were somewhat confident, and 73 (7.6%) were not confident at all (Table 3).

**Table 3:** Health service related characteristics of patients at emergency departments in Gondar University Referral Hospital, Northwest Ethiopia, May 2012

**Health service related character**	**Number of patients**	**Percent**
**Cancelled hospital visit last time while needed to do so**		
Yes	169	17.6
No	792	82.2
Don’t remember	2	0.2
**Reason for cancelling visit before (n = 169)**		
Lack of money	92	54.4
Other reason	67	39.7
I don’t know	10	5.9
**Degree of confidence to get good service in the future**		
Very confident	582	60.4
Confident	268	27.8
Somewhat confident	40	4.2
Not confident at all	73	7.6
**Believed discriminated**		
No	257	26.7
Yes	706	73.3

**Figure 2:**
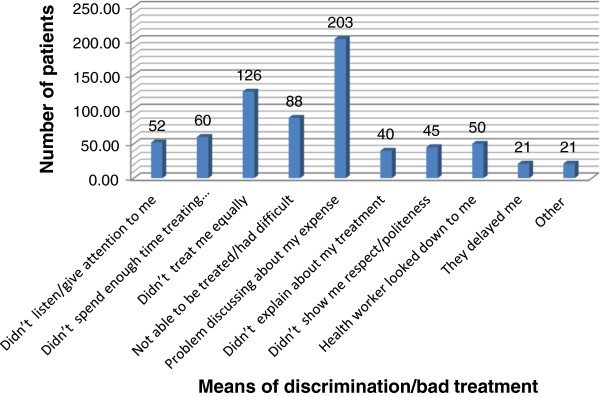
Means of discrimination/bad treatment among patients at emergency departments in Gondar University Referral Hospital, May 2012.

A large number, 706 (73.3%) 95%CI: 70.4%-76.1%, of patients reported that they had been discriminated against or treated badly during the provision of service in the hospital. The major source of discrimination and bad treatment were failure of the health worker to discuss the expenses for treatments, 203 (28.7%), unequal handling or treatment by the health workers, 126 (17.8%), inability to get treatment, 88 (12.5%), not spending enough time with patient, 60 (8.5%), not listening to the patient 52 (7.4%), looking down on the patient, 50 (7.1%), and the health worker talking unkindly to the patient, 7 (1.0%) (Figure 2).

### Overall quality of care/patient satisfaction

#### Reliability analysis

To check for the internal consistency of the 20-item satisfaction measurement tool, we did reliability analysis. The overall Cronbach’s alpha value was 0.88 reflecting a very high consistency of instrument-to-measure the satisfaction. The inter-item correlation was also low (all well below 0.6) showing that each item was measuring distinct characteristics of patient satisfaction. On top of the overall alpha value, the value for each of the 20 items was also high (Table 4).

**Table 4:** Reliability analysis of the 20 patient satisfaction measurement items among patients at emergency departments in Gondar University Referral Hospital, Northwest Ethiopia, May 2012

**Item**	**Mean score**	**Standard deviation**	**Cronbach’s α**
Courtesy of staff in the registration area	3.55	1.09	0.877
Comfort and pleasantness of the waiting area	3.31	0.99	0.875
Comfort and pleasantness during examination	3.40	1.01	0.873
Friendliness/courtesy of the nurse	3.60	0.91	0.873
Concern the nurse showed for doing medical orders	3.68	0.87	0.876
Courtesy of security staff	3.49	0.95	0.876
Courtesy of staff who transfer the patients	3.50	0.97	0.876
Length of wait before going to an exam room	3.35	1.17	0.877
Friendliness/courtesy of the care provider	3.73	0.84	0.872
Explanations the care provider gave you about your condition	3.02	1.11	0.875
Concern the care provider showed for your questions or worries	3.35	1.02	0.871
Care provider’s efforts to include you in decisions about your treatment	3.06	1.09	0.872
Information the care provider gave you about medications	2.94	1.08	0.876
Instructions the care provider gave you about follow-up care	3.01	1.05	0.875
Degree to which care provider talked with you using words you could understand	3.83	0.84	0.875
Amount of time the care provider spent with you	3.58	0.92	0.872
Frequency of being visit by physicians	3.46	0.99	0.872
Overall cheerfulness of hospital practice	3.50	.99	0.874
Overall cleanliness of hospital practice	3.40	1.05	0.881
Likelihood of your recommending the practice to others	3.81	1.00	0.875
**Total items score**	**3.43**	**1.00**	**0.88**

The overall satisfaction using the mean score indicated that 498 (51.7%) 95%CI: (48.4% - 54.9%) were satisfied with the service, the providers and the facility suitability whereas 465(48.3%) 95%CI: (45.1%- 51.6%) were dissatisfied. Assessing the clients’ satisfaction for each item, 36.3% of the clients were dissatisfied or indifferent to the courtesy of staff in the registration area while 63.7% were either satisfied or very satisfied. In about two-thirds (64.2%) of clients, the information provided about medication was not satisfying and ranked fair or below. The degree to which the care providers talked to the patient using words which the patients could understand was high at 74.2% (Table 5).

**Table 5:** Levels of satisfaction based on 20 measurement items among patients visiting emergency departments in Gondar University Referral Hospital, Northwest Ethiopia, May 2012

**Item**	**Level of satisfaction**				
	**Very unsatisfied**	**Unsatisfied**	**Fair**	**Satisfied**	**Very satisfied**
	**No. (%)**	**No. (%)**	**No. (%)**	**No. (%)**	**No. (%)**
Courtesy of staff in the registration area	62 (6.4)	112 (11.6)	176 (18.3)	464 (48.2)	149 (15.5)
Comfort and pleasantness of the waiting area	35 (3.6)	185 (19.2)	269 (27.9)	398 (41.3)	76 (7.9)
Comfort and pleasantness during examination	27 (2.8)	192 (19.9)	215 (22.3)	429 (44.5)	100 (10.4)
Friendliness/courtesy of the nurse	32 (3.3)	79 (8.2)	240 (24.9)	501 (52.0)	111 (11.5)
Concern the nurse showed for doing medical orders	20 (2.1)	75 (7.8)	224 (23.3)	520 (54.0)	124 (12.9)
Courtesy of security staff	38 (3.9)	105 (10.9)	258 (26.8)	468 (48.6)	94 (9.8)
Courtesy of staff who transfer the patients	29 (3.0)	116 (12.0)	289 (30.0)	399 (41.4)	130 (13.5)
Length of wait before going to an exam room	78 (8.1)	175 (18.2)	189 (19.6)	378 (39.3)	143 (14.8)
Friendliness/courtesy of the care provider	18 (1.9)	66 (6.9)	196 (20.4)	560 (58.2)	123 (12.8)
Explanations the care provider gave you about your condition	99 (10.3)	229 (23.8)	249 (25.9)	325 (33.7)	61 (6.3)
Concern the care provider showed for your questions or worries	40 (4.2)	175 (18.2)	254 (26.4)	394 (40.9)	100 (10.4)
Care provider’s efforts to include you in decisions about your treatment	77 (8.0)	242 (25.1)	257 (26.7)	316 (32.8)	71 (7.4)
Information the care provider gave you about medications	102 (10.6)	247 (25.6)	270 (28.0)	299 (31.0)	45 (4.7)
Instructions the care provider gave you about follow-up care	76 (7.9)	246 (25.5)	286 (29.7)	301 (31.3)	54 (5.6)
Degree to which care provider talked with you using words you could understand	19 (2.0)	40 (4.2)	202 (21.0)	523 (54.3)	179 (18.6)
Amount of time the care provider spent with you	24 (2.5)	97 (10.1)	258 (26.8)	465 (48.3)	119 (12.4)
Frequency of being visit by physicians	38 (3.9)	131 (13.6)	253 (26.3)	431 (44.8)	110 (11.4)
Overall cheerfulness of hospital practice	44 (4.6)	101 (10.5)	261 (27.1)	442 (45.9)	115 (11.9)
Overall cleanliness of hospital practice	51 (5.3)	159 (16.5)	209 (21.7)	442 (45.9)	102 (10.6)
Likelihood of your recommending the practice to others.	42 (4.4)	62 (6.4)	156 (16.2)	477 (49.5)	226 (23.5)

### Determinants of patient satisfaction on emergency medical care

In the multiple logistic regression analysis using backward stepwise method, the OPD site visited was significantly associated with level of satisfaction (p < 0.0001). Individuals who visited OPD 2 were 1.6 times more likely to be satisfied with the service as compared to those served at OPD 5 (AOR = 1.6, 95%CI:1.1, 2.4). Patients who visited OPD 3 were 3.4 times more likely to be satisfied with the emergency service when compared to those visiting OPD 5 (AOR = 3.4, 95%CI: 2.1, 5.8).

The visiting days also had an effect on satisfaction of patient with emergency care provided (p < 0.05). Patients who arrived on Monday were less likely to be satisfied when compared to those visiting on Sundays, even though this turned out to be non-statistically significant in the final model. Patients who came to OPD on Thursday (AOR = 1.7, 95%CI: 1.1, 3.0) and Friday (AOR = 1.9, 95%CI: 1.1, 3.4) were more likely to be satisfied when compared to their counterparts arriving on Sunday.

The medical condition on arrival was a predictor for patient satisfaction at the emergency department (p < 0.0001). Patients who were very sick on clinical assessment on their arrival were 3.6 times more likely to be satisfied when compared to those with good conditions (AOR = 3.6, 95%CI:2.3, 5.5) on arrival and patients with moderate condition were 1.6 times more likely to be satisfied with the service (AOR = 1.6, 95%CI:1.1, 2.5). Patients very confident with the service provided were nearly twice more likely to be satisfied with emergency service (AOR = 1.9, 95%CI: 1.1, 3.1).

Another vital determinant of patient satisfaction was perception of being discriminated against by health care providers. Those who felt that they were discriminated against at some care provision point were 2.5 times less likely to be satisfied with the service (AOR = 0.4, 95%CI: 0.2, 0.7). Patient satisfaction was not significantly associated with waiting time, residence, gender, age or other variables (Table 6).

**Table 6:** Logistic Regression analysis of factors associated with patient satisfaction among emergency outpatient departments in GURH, Northwest Ethiopia; May 2012

**Character**	**Level of satisfaction**		**OR (95% C.I.)**		**P-value**
	**Satisfied**	**Not satisfied**	**Crude**	**Adjusted**	
OPD site visited					<0.0001
Medicine	201	215	1.3 (0.9,1.8)	1.3 (0.9, 1.2)	.164
Surgery	126	106	1.6 (1.1, 2.4)	1.6 (1.1, 2.4)	.039
OBGY	87	38	3.1 (1.9, 5.0)	3.4 (2.1, 5.8)	<0.0001
Oral health, psychiatry	13	11	1.6 (0.7, 3.7)	1.5 (0.6, 3.8)	.379
Pediatrics	71	95	1	1	
Visiting days of the week					.049
Monday	50	65	0.9 (0.5, 1.5)	.9 (0.5,1.5)	.587
Tuesday	103	92	1.3 (0.8, 2.1)	1.4 (0.8, 2.3)	.231
Wednesday	97	93	1.2 (0.7, 1.9)	1.4 (0.8, 2.3)	.223
Thursday	91	67	1.5 (0.9, 2.6)	1.7 (1.1, 3.0)	.044
Friday	64	49	1.5 (0.9, 2.6)	1.9 (1.1, 3.4)	.027
Saturday	48	48	1.1 (0.6, 2.0)	1.2 (0.6, 2.1)	.636
Sunday	45	51	1	1	
Medical condition on arrival					<0.0001
Good condition	45	80	1	1	
Somewhat sick	185	231	1.4 (0.9, 2.2)	1.6 (1.1,2.5)	.030
Very sick	268	154	3.1 (2.0, 4.7)	3.6 (2.3, 5.5)	<.0001
Degree of confidence on the hospital					<0.0001
Very confident	342	240	1.7 (1.1, 2.8)	1.9 (1.1,3.1)	.019
Confident	109	159	0.8 (0.5, 1.4)	0.95 (0.6, 1.7)	.853
Somewhat confident	14	26	0.7 (0.3, 1.5)	0.6 (0.3, 1.3)	.200
Not confident at all	33	40	1	1	
Reported discrimination in service					
No discrimination	172	85	1	1	<0.0001
Felt discriminated	326	380	0.4 (0.3, 0.6)	0.4 (0.2,0.7)	

## Discussion

The study assessed the quality of health service at the emergency units of Gondar University Referral Hospital using a 20-item patient satisfaction questionnaire. Many of the patients presented from Monday to Wednesday accounted to more than 60% of visitors in all days of the week. Many of the patients reported at the beginning of the week similar to the situation in other studies [7].

Injuries were the leading cause of emergency OPD visits with 14.5% of all visitors. This is in line with studies in different countries [6-8]. The higher number of injuries in emergency OPD in this study was due to significant road traffic and vehicle crashes that caused many patients to visit the emergency department. Public violence among men and domestic violence with women was also a common cause of injuries [8]. Other unusual problems observed in the emergency diagnoses were non-communicable diseases including cancer, cardiovascular diseases, hematologic, mental illness, metabolic and neurologic disorders and these contributed to a total of 18.2%. This confirms the threat of a double burden of infectious and non-communicable disease that will be a challenge to the health care system in Ethiopia [2,24]. Cardiovascular disorders alone contribute 5.7% of emergency visits which supports previous evidence to the rise of the problems and signals that the emergency care at hospitals and referral system has to be revised based on the current prevailing conditions [9].

Discrimination and bad treatment of patients was found to be high (73.3%) in this study mainly due to poor interpersonal communication of patients’ problems, their treatment and/or cost of care. This tells clinicians that investigation and prescriptions are not the only needs of patients and all the concerns should be addressed in an understandable way. Other studies also identified that physician and nurse communications with patients were important determinants of satisfaction [18].

The reliability analysis for the patient satisfaction measurement items resulted in an overall Cronbach’s alpha score of 0.88. This tells that the tool is consistent for measuring the patient satisfaction. Moreover, the inter- item correlation coefficients were all less than 0.05 showing the items are mutually exclusive and measure different issues.

The level of patient satisfaction of 51.7% with the emergency service at the hospital is very low. This figure is lower than studies in Iran and other countries [16,18] where there is 63% or more satisfaction with emergency care. The reason may be due to the presence of a specialized emergency care service in the later that provided a better care for patients. It is however, much higher than the findings from Pakistan [17]. The very high discrepancy is due to the fact that the Pakistan study additionally considered the ambulance service call for emergency conditions to reach health facilities that might have overestimated the dissatisfaction. This will have a negative effect on health care utilization on top of a very low rate. It is vital to save lives of patients by improving the suitability and quality of care to patients.

Patients who were served in OBGY and Surgery departments were 1.6 and 3.44 times more likely to be satisfied as compared to those visiting other departments respectively. This may be related to the nature of intervention that solves the patients’ concerns and observed illness via surgical and other procedures.

Patients who arrived on Mondays were less likely to be satisfied as compared to those arriving Sunday. This is due to the overstretched OPD by the large numbers of regular and emergency patients that makes hard to adequately treat all patients satisfactorily.

Patients with serious medical condition were nearly 3.6 times more likely to be satisfied with the service as compared to those with good condition. This is probably due to the better attention and more time given to critical patients than those in good condition. Senior physicians also are more likely to be consulted for this group of patients leading to better handling. Critical patients’ are also given priority at the gate and may not be subjected to bad treatment or discrimination. This is because of the lack of classification of patients on arrival and provide care accordingly that would improve patient handling [20,21].

Having felt discriminated is a negative determinant of satisfaction in the emergency care. Those who felt that they were badly treated were 2.5 times less likely to be satisfied with the service than their counterparts.

### Study limitations

The study participants’ selection depended on those reporting to emergency departments and might have dealt with more critical cases. The study involved interview of patients and care takers in case of critical conditions to the patient and this might result in minor differences in response regarding quality of care of the patient. As this is a cross-sectional study, the pattern of all disease could not have been viewd over time. The patient satisfaction might have been influenced by surgical interventions done to some in need against those medically treated.

## Conclusions

Injuries and non-communicable diseases emergency as cardiovascular emergencies are very common among patients in Northwest Ethiopia.

There is low level of patient satisfaction in the emergency department. The sources of dissatisfaction were lack of courtesy of staff, physical discomfort, unavailability of drugs, under treatment of patient not in serious medical condition, and discrimination and bad treatment of patients.

Type of OPD visited, days of visit, medical condition on arrival, confidence on the hospital to get good treatment, and presence of discrimination/bad treatment of patients were statistically significantly associated determinants of patient satisfaction.

Hospitals shall prepare themselves to address the increasing challenge of non-communicable disease emergencies that would result in longer duration of stay, high cost of care, and increasing hospital mortality. There has to be a mechanism to motivate staff to handle patients of all categories of severity properly and equally without discrimination and bad treatment. There is a need for evidence-based interventions in emergency care services in physician care, nursing care, courtesy of staff, physical comfort, and equal treatment to improve satisfaction. Hospitals shall improve patient services to narrow the gap between health coverage and utilization.

## Competing interests

This research was sponsored by the University of Gondar; however the sponsorship has no influence or linkage to the findings or publication of this manuscript. The authors declare that there are no competing interests.

## Authors’ contributions

BWT, MOY, and ZTK were involved in the concept, design, data collection and analysis. All authors read and approved the final manuscript.

## Pre-publication history

The pre-publication history for this paper can be accessed here:

http://www.biomedcentral.com/1471-227X/14/2/prepub
